# Special Issue: Mass Spectrometric Proteomics

**DOI:** 10.3390/molecules24061133

**Published:** 2019-03-21

**Authors:** Paolo Iadarola

**Affiliations:** Department of Biology and Biotechnologies “L.Spallanzani”, University of Pavia, 27100 Pavia, Italy; piadarol@unipv.it

The term “Proteomics” refers to the characterization of the proteome, that is, all proteins present in a biological system. Because the protein content of an organism changes in response to many conditions, the proteome dynamism helps investigation on differences in protein states between different conditions. Identifying all proteins present in biological systems is a difficult task and a crucial role in enabling the analysis of proteomes is played by mass spectrometry (MS). Given the complexity of biological systems, high performance liquid chromatography-based (HPLC) separations are currently applied to separate the mixture of proteins before analysis. After these extensive procedures, confident identifications may be obtained by MS and tandem MS (MS/MS) either on intact proteins (top-down) or on peptides obtained after enzymatic digestion (bottom-up). Changes in protein abundances between different biological states may be obtained by several approaches, with or without stable isotopic labeling. 

In this Special Issue of *Molecules* the reader will find a topical selection of both research and review articles which bring together different sophisticated methodological strategies with applications in protein profiling of organs, in the study of post translational tissue modifications or in the investigation of the molecular mechanisms behind human disorders. 

Mauri and co-workers [1] open the section of seven research articles with a study that describes the application of multidimensional protein identification technology (MudPIT) to discover the urinary proteomes of head and neck squamous cell carcinoma (HNSCC) and thyroid cancer patients. They analyzed urine samples from these subjects to identify protein profiles prior and after infusion with ^10^Boron and neutron capture therapy (BNCT) during surgery. The results of this study allowed the identification of several inflammation- and cancer-related proteins, which could be potential tumor biomarkers. In particular, a reduction of three proteins (Galectin-3 binding protein, CD44, and osteopontin) that were involved in inflammation was observed after treatment. Analysis of the urinary proteome during and after therapeutic interventions made it clear that this fluid reflects changes that are induced by several diseases, including different types of cancer. 

Another interesting study from the same research group aimed at exploring the potentiality of shotgun proteomic platforms in the characterization of the status and the evolution of metabolic pathways during ex vivo lung perfusion (EVLP), an emerging procedure that allows organ preservation in lung transplantation. The application of a nanoLC–MS/MS system to the proteome analysis of lung tissues from three experimental groups (native, pre- and post-EVLP) of an optimized rat model allowed identification of potential EVLP-related biomarkers. Given the promising findings provided by this work, future perspectives will attempt to confirm these data with the aim of translating the experimental research to human specimens [2]. 

The huge potential of sophisticated technologies in the proteomic area is shown by two original articles.

The report by Delphine and coworkers [3] demonstrates the utility of quadrupole time-of-flight (Q–ToF) hybrid mass spectrometer equipped with electrospray ionisation source (ESI). Their study aimed at optimising tandem MS analysis by testing the effect of some of the parameters concerning the in-source collision-induced dissociation (IS-CID) procedure in combination or not with conventional CID. A total of 11 MS/MS methods were assessed on samples bearing increased complexity, including individual milk protein standards, mixed protein standards and cow’s raw milk samples from different breeds. Results of top-down sequencing from the nine most abundant proteoforms of caseins, alpha-lactalbumin and beta-lactoglubulins were compared. Nine MS/MS methods achieved more than 70% sequence coverage overall to distinguish between allelic proteoforms varying only by one or two amino acids. 

Zhang and colleagues [4] used ultra-performance liquid chromatography/Orbitrap Fusion mass-spectrometry (UPLC/MS/MS) and chemometrics to study *Ophiocordyceps sinensis*, an important fungal drug used in Chinese medicine. This approach allowed identification of a number of marker tryptic peptides in the wild *O. sinensis* fruiting body and in various commercially available mycelium fermented powders. This represented the first extensive study on the authentication of *O. sinensis* and cultured *Ophiocordyceps* mycelia.

The isobaric tags for relative and absolute quantitation (iTRAQ) technique was used by Su and coworkers [5] to investigate the oviduct of *Rana chensinensis* (another important traditional Chinese medicine resource widely used in the treatment of asthenia after sickness or delivery, deficiency in vigor, palpitation and insomnia), which, in contrast to the breeding period, significantly expands during prehibernation. To explain this phenomenon at the molecular level, the protein expression profiles of *Rana chensinensis* oviduct during these two conditions were analyzed. Among the 4479 proteins identified, 312 were up- or down-regulated between these two periods. The application of gene ontology (GO) and Kyoto Encyclopedia of Genes and Genomes (KEGG) analyses allowed them to understand that this distinctive physiological phenomenon of *Rana chensinensis* oviduct was mainly involved in extracellular matrix–receptor interaction, metabolic pathways, and focal adhesion. 

The interest of Li and co-authors [6] was focused on the study of protein lysine acetylation (PKA), a key post-translational modification involved in the regulation of various biological processes in rice. They applied an MS-based label-free approach to perform a quantitative analysis of PKA proteins in rice callus, root, leaf and panicle. The identification of 890 PKA proteins, which covered 1536 sites, allowed construction of a tissue atlas of rice acetylome and provided an overall view of the acetylation events in rice tissues. 

The targeted proteome analysis performed by Tokumaru and co-workers [7] aimed at investigating the SigE dependent-regulation of central metabolism in *Synechocystis sp.* PCC 6803, a model cyanobacteria. In their study, they compared the protein abundance profiles among the wild type, a SigE deletion mutant (DSigE), and a SigE over-expression (SigEox) strain. The protein profile showed that SigE plays a pivotal role as a positive regulator of oxidative pentose phosphate pathway (OxPPP) activity and NADPH reproduction. The results also suggested that SigE over-expression increases GdhA abundance, which is involved in the nitrose assimilation pathway using NADPH and downregulates the proteins involved in photosynthesis.

The Special Issue is concluded by a compilation of review articles on different topics. 

Given the relevance of isobaric labeling reagents for quantitative isobaric derivatization strategies in proteomics, Bachor and colleagues [8] review the trends in the design of new isobaric markers. The development of selective methods for introducing into a peptide a tag that increases the multiplexicity of markers and the sensitivity of measurement and lowers the cost of synthesis is currently a topic of great interest in proteomics. Different types of isobaric reagents are used in quantitative proteomics, and their chemistry and advantages offered by their application are clearly presented in this article. 

In recent years, platinum-based anticancer drugs (especially cisplatin) have played an important role in the clinical chemotherapy of cancer. Due to the adverse effects and acquired resistance of these drugs, efforts have been made to exploit novel anticancer metallodrugs and unveil the molecular mechanism of anticancer activity and drug resistance. The article by Jia and co-workers [9] is focused on the identification, by MS-based quantitative strategies, of proteins which specifically respond or bind to metal-based anticancer drugs and on the elucidation of their mechanisms of action.

Finally, Zdenek Perutka and Marek Šebela [10] review the cleavage properties of bovine pseudotrypsin, a trypsin proteoform, in the digestion of protein samples with a different complexity. While cleaving peptide bonds predominantly at the conventional trypsin cleavage sites, pseudotrypsin shows non-specific cleavages (mostly after the aromatic residues of Tyr and Phe) which are not expected to occur for the major trypsin forms. The valuable information provided by this peculiar activity could be of great utility in common proteomics protocols. 

I hope that the content of this Special Issue meets the expectations of readers and would like to thank all authors for their excellent contributions.
